# A young man with Guillain–Barré syndrome, with a stroke-like presentation and physical findings suggestive of cervical myelopathy

**DOI:** 10.5339/qmj.2023.16

**Published:** 2023-08-08

**Authors:** Maria Siddiqi, Sundus Sardar, Muhammad Ibrahim Alhatou

**Affiliations:** ^1^Department of Internal Medicine, Neurology Section, Alkhor Hospital, Hamad Medical Corporation, Doha, Qatar E-mail: msiddiqi2@hamad.qa ORCID: http://orcid.org/0000-0002-8092-0346; ^2^Department of Internal Medicine, Hamad Medical Corporation, Doha, Qatar

**Keywords:** Guillain–Barré syndrome, myelopathy, os odontoideum

## Abstract

We describe the case of a 44-year-old gentleman with hypertension and asthma presenting to the emergency department after noticing right upper-extremity weakness upon awakening. Brain imaging did not reveal a stroke. Initial neurological examination pointed to cervical myelopathy with radiculopathy as well as possible underlying length-dependent peripheral neuropathy as there was right arm strength of 4/5 and there were brisker (3+) reflexes all over except at the right biceps reflex and both ankle reflexes. Cervical spine magnetic resonance imaging (MRI) showed myelomalacia at the C2 level and an os odontoideum (OO). Os odontoideum is a chronic condition that occurs due to the failure of the center of ossification of the dens to fuse with the body of C2. By the next day after a few hours of sustaining a fall, weakness progressed to quadriparesis, without a sensory level on examination, followed by urinary retention. This situation was attributed to a possible cervical cord contusion due to the fall in the presence of OO, with other possibilities being spinal cord hemorrhage, infarct and transverse myelitis. However, repeat scanning of the cervical spine (MRI) did not reveal any acute cord changes. The initial examination for common causes of peripheral neuropathy did not reveal any findings. Finally, the diagnosis of Guillain–Barré syndrome (GBS) was considered, and treatment was initiated with intravenous immunoglobulin. Cerebrospinal fluid analysis was normal. The diagnosis was confirmed using electromyography. Our patient’s initial presentation of monoparesis and progression in an asymmetric descending manner was unusual for GBS. His initial presentation mimicked a stroke, and the later progression masqueraded as cervical myelopathy secondary to a chronic cervical cord lesion. The presence of a cervical cord lesion (upper motor neuron) concealed the expected areflexia in GBS. The presence of OO on spine imaging, absence of expected areflexia in GBS, and progression to paraparesis after the fall sidetracked the direction of the initial investigation and led to a relative delay in diagnosis. Nonetheless, appraising the diagnostic data in the clinical context led to an appropriate diagnosis. We emphasize the importance of reconciling the available clinical and diagnostic information to reach the correct diagnosis.

## Introduction

Guillain–Barré syndrome (GBS) is an acute neuropathy of autoimmune etiology that classically presents as a symmetric ascending flaccid weakness of the lower limbs that may extend to involve the upper limbs, diaphragm, and cranial nerves over hours to days with or without sensory and autonomic symptoms. The weakness of the diaphragm can cause respiratory compromise. About 20–40% of patients require ventilator support. The involvement of cranial nerves can cause facial weakness, dysphagia, and dysphonia, whereas autonomic involvement may cause blood pressure lability, cardiac arrhythmias, abnormal sweating, and bladder dysfunction. The symptoms can progress up to 4 weeks.^1,2^ This is followed by a period of stability and subsequent recovery. Areflexia or hyporeflexia is almost universally present. Several variant forms of GBS are known. These are based on clinical and pathologic features. Acute inflammatory demyelinating polyneuropathy is the most common form of GBS. There are acute axonal neuropathies with acute motor axonal neuropathy (AMAN) and acute motor and sensory axonal neuropathy. In addition, there are other uncommon variants such as pure sensory GBS, pandysautonomia (predominantly affecting the autonomic nervous system), acute bulbar palsy, and the GQ1b antibody syndromes. The GQ1b syndromes include the Miller Fisher syndrome, Bickerstaff brainstem encephalitis, and pharyngeal-cervical-brachial variant. These present predominantly with cranial nerve abnormalities.^3–10^ About two-thirds of the patients provide a history of a preceding gastrointestinal or respiratory infection, 1–6 weeks before the onset of symptoms.^11^ GBS has been linked to vaccinations, including influenza and COVID-19 vaccines, but the evidence to prove causality remains weak.^12–17^ The standard treatment options include intravenous immunoglobulin (IVIg) and plasmapheresis which can be used alternatively as they are equally effective.^18^ We report a case of GBS from a tertiary care hospital in Qatar, of a patient who initially presented with monoparesis and progressed to quadriparesis in a descending manner as opposed to a typical ascending weakness presentation. The publication of this case was approved by the Institutional Ethics Committee (MRC-04-22133), and consent was obtained from the patient.

## Case Presentation

**Figure 1.**
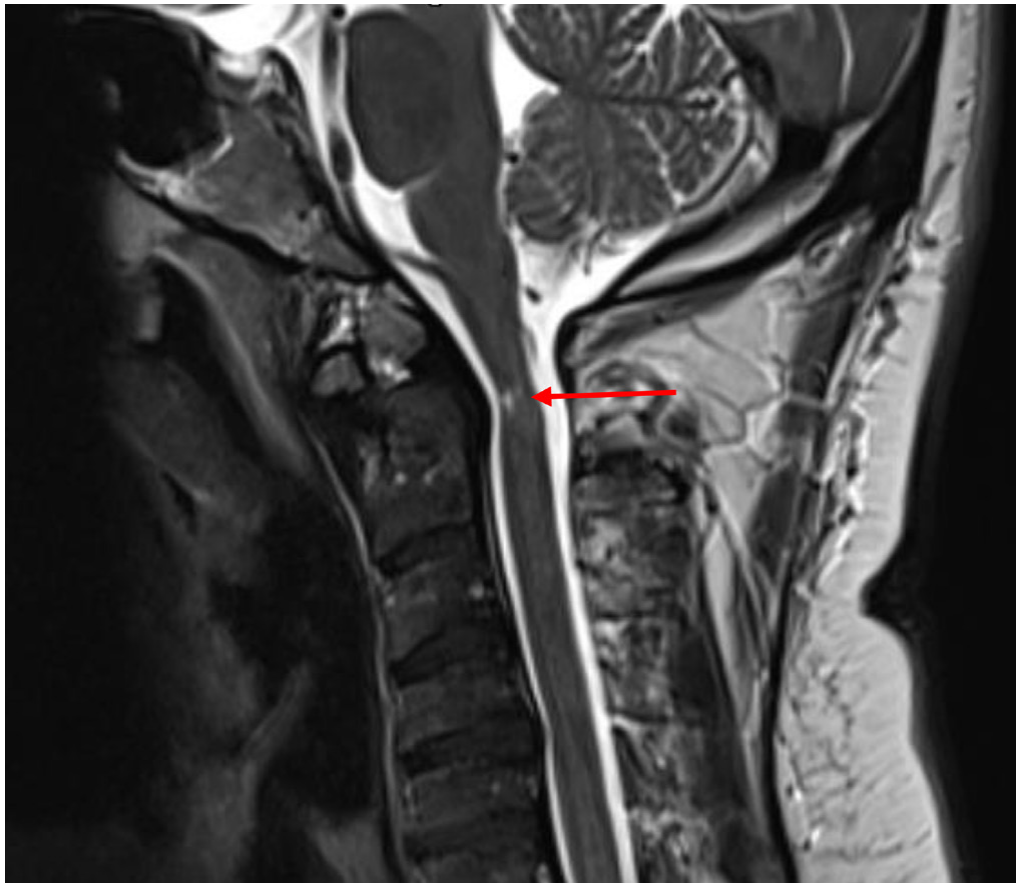
Focal myelomalacia and atrophy (arrow) involving the cervical spinal cord at the C2 level

**Figure 2.**
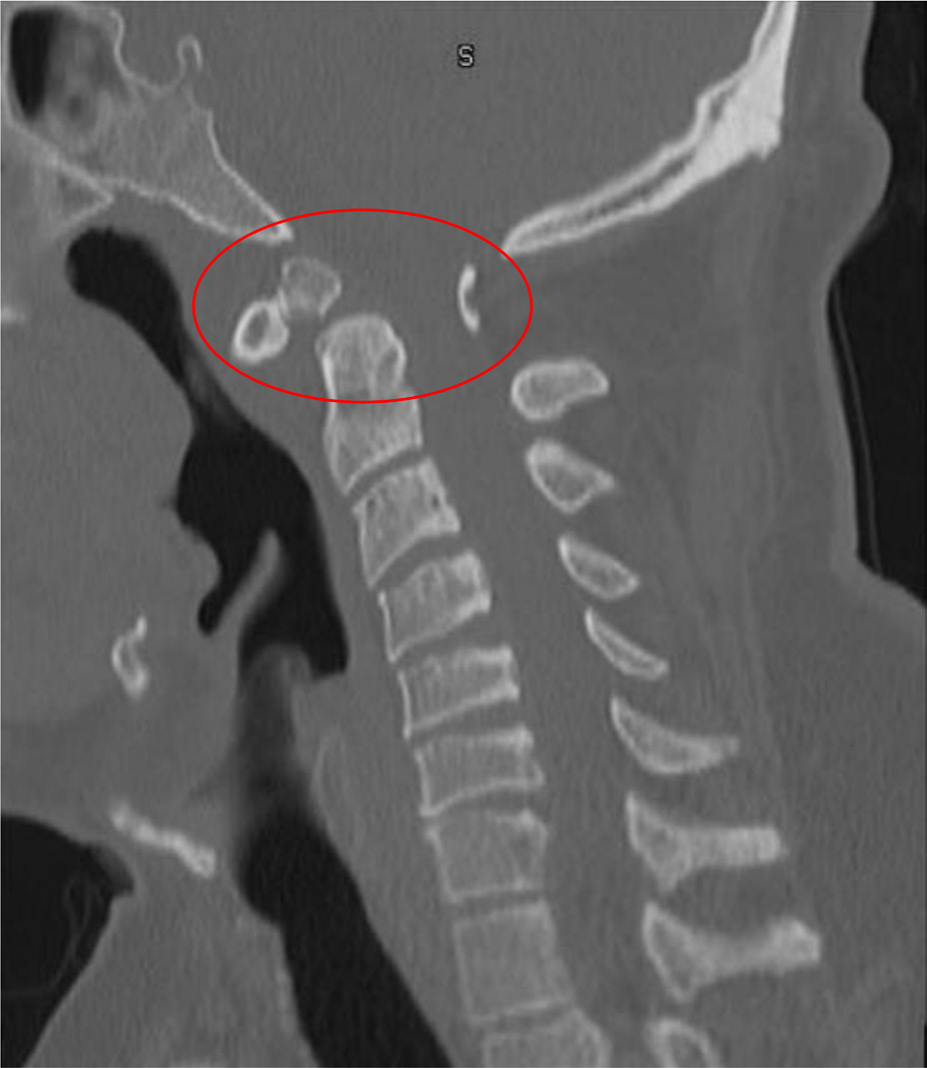
Corticated bony fragment in relation to the hypoplastic dens of C2 vertebra, suggesting os odontoideum with a narrowed spinal canal at the same level (red circle)

A 44-year-old gentleman with hypertension and bronchial asthma presented to the emergency department, with weakness of the right upper extremity discovered after awaking. Facial deviation, sensory symptoms, or dysarthria were absent. He was found to have weakness of the right arm (4/5) and was suspected of having a stroke. Computed tomography (CT) of the head and CT angiogram of cervical and cranial circulation were unremarkable. He was admitted for further investigation. Aspirin and atorvastatin were commenced. The next day, neurology service was consulted. Neurological examination revealed normal sensorium, speech, language function, and cranial nerves. The tone was normal all over, and the strength was normal in both lower limbs and the left upper limb. The right upper-limb strength at the shoulder and elbow was 4/5. The wrist and hand grip were 3/5. The right triceps and both ankle reflexes were absent. All other reflexes were 3+ without clonus. Plantar responses were equivocal. Coordination and sensory examinations were normal. Magnetic resonance imaging (MRI) of the head, including diffusion-weighted imaging, was also unremarkable. We could not localize this presentation to a single lesion. The brisker reflexes in all extremities sparing the jaw jerk with normal cranial nerve examination suggested a cervical cord lesion. The right-arm weakness up to the deltoid muscle localized the proximal extent of the cord lesion to the C5-6 segment or above. The right triceps areflexia could be explained by an accompanying right C6-7 root compression; however, this could still not explain ankle areflexia unless there was an underlying peripheral neuropathy. Cervical myeloradiculopathy can be attributed to compressive or non-compressive etiology.^10–14^ There were a few shortcomings in making a case of cervical myelopathy. First, the onset of right-arm weakness was acute, but the presence of brisker reflexes pointed to a chronic process involving the cervical spinal cord. Also, one could argue that in the absence of clonus, the 3+ reflexes were not pathological. Second, sphincter dysfunction and a sensory level were absent. These findings did not point to the diagnosis of acute cervical myelopathy. The MRI of the cervical spine revealed os odontoideum (OO), with the posterior aspect of the cranial part of the C2 vertebra indenting the spinal canal. The cervical spinal cord at that level showed focal thinning and noncontrast-enhancing focal T2-hyperintense and T1-hypointense signals, suggesting myelomalacia (Figure 1). The initial work-up for peripheral neuropathy, including serum TSH, HbA1c, and vitamin B12, did not reveal any abnormality. The next morning, while trying to get out of bed, the patient sustained a fall. The same evening, he reported weakness in all limbs without involvement of sphincters. The examination revealed quadriparesis with predominant weakness of distal muscles, with an overall strength of 3–/5 proximally and 1/5 distally in the upper extremities and 3+/5 proximally and 2/5 distally in the lower limbs. The remaining examination was unchanged. The quick progression of the weakness after the fall prompted the consideration of an acute cervical cord injury-spinal contusion in relation to the OO. Other possibilities were spinal cord infarction or hemorrhage and less likely transverse myelitis. A CT scan of the cervical spine was obtained that evening and did not reveal any additional abnormalities except OO (Figure 2). We planned a spinal tap that the patient did not consent to, until the next day. To address the possibility of transverse myelitis, one dose of 1 g of IV methylprednisolone was administered. Atorvastatin and aspirin were stopped. MRI, full spine with contrast and diffusion-weighted images, was requested, which did not reveal any of the suspected conditions. Neurosurgeon was consulted who did not deem the cord lesion to be responsible for the presentation or progression. The clinical scenario highly indicated GBS. The patient was commenced on IVIg that evening, and methylprednisolone was discontinued. Cerebrospinal fluid analysis was normal. Serum protein electrophoresis did not show monoclonal bands. On day 4, he developed urinary retention and required the placement of a Foley catheter. Urinary retention was attributed to dysautonomia of GBS. Electromyography was performed, which revealed AMAN. The next day, neurological examination revealed the progression of the weakness of the left lower limb from 2/5 to 1/5 distally, and the knee reflexes were observed to be diminished (1+). He received 2 g/kg of IVIg over a period of 5 days. His strength improved to 3–/5 on the right leg and 2/5 on the left distally. On further inquiry, he reported a diarrheal illness a week ago, as well as vaccination against influenza 2 weeks prior to the onset of symptoms. After 3 days, he regained normal micturition function. He was transferred to the rehabilitation center where he stayed for 3 months. Since then, he has been receiving outpatient physiotherapy to date. At a 10-month post-discharge follow-up by teleconsultation, he reported mostly being wheelchair bound, being able to take a few steps with the help of a walker and eating independently using occupational self-feeding tools.

## Discussion

Os odontoideum and myelomalacia are chronic conditions that typically do not present with acute onset and progression of motor weakness. It is a condition originally thought to be congenital in nature due to the failure of the center of ossification of the dens to fuse with the body of C2. Another mechanism, trauma causing an unrecognized fracture through the dens’s growth plate during early childhood, has also been proposed. It can lead to abnormal mobility of the dens with respect to C2. Radiologically, it is defined as an oval- or round-shaped ossicle, with smooth circumferential cortical margins representing a hypoplastic odontoid process (dens) that has no continuity with the C2 vertebral body. Typically, it is asymptomatic but can rarely present with neck pain or symptoms of cervical myelopathy caused by excessive and abnormal motion between the C1 and C2 vertebrae, resulting in stretching or compression of the spinal cord by the OO.^19–23^ This scenario seemed likely in our patient. If the OO caused acute myelopathy due to trauma from the fall, we still did not have an explanation for the initial monoparesis and the distal lower-limb areflexia. Also, we did not find an acute cervical cord lesion after the fall. To add to the complexity of the case, his symptoms were also not typical of GBS. The GBS typically presents as a symmetric ascending paralysis with facial weakness and hyporeflexia. Some other features can variably be present depending on the variant.^3–10,11^ Our patient’s initial presentation of monoparesis and progression in an asymmetric descending manner was unusual for GBS. Predominant or initial weakness of upper limbs in GBS is rarely described in the literature with other variants.^24–26^ To our knowledge, it has not been described in the AMAN variant that our patient had. Moreover, the cervical cord lesion and the absence of areflexia at the level of motor involvement led to a delay in the consideration of this diagnosis. Initially, it mimicked an acute stroke, and later the finding of the cervical cord lesion served as a red herring in the context of progression to quadriparesis. It distorted the clinical scenario and sidetracked the direction of initial examination and management. In this case, the combination of the upper and lower motor neuron signs in the limbs stemmed from two different pathologies: the cervical cord lesion and the acute motor neuropathy. The upper motor neuron lesion (C2 cord) concealed areflexia in our patient, which would be expected in GBS. Explaining all the symptoms and signs assigning a unifying diagnosis is widely taught and is the best practice but not always possible.

## Conclusion

Atypical presentations of different neurological conditions, including GBS, are likely to occur. Diagnosing such patients can be difficult and tedious. However, it is crucial to diagnose GBS early in its course, as its progression to respiratory failure and development of autonomic dysfunction can lead to labile hypertension and arrhythmias, which can contribute to mortality in GBS. Early diagnosis can provide an opportunity for timely intervention, including ventilator support and management of autonomic dysfunction.^27–29^ These measures can be life-saving. The clinical presentation of any neurological case should not be attributed to any lesion found in brain or spinal cord imaging unless the symptoms’ onset and progression as well as the examination findings can be explained by the lesion itself. Incidentally diagnosed lesions of the spinal cord or brain on scans can mislead the course of investigation and management. The aim of presenting this case is to highlight the importance of scrutinizing the results of investigations in the context of the clinical scenario rather than evaluating and addressing them in isolation.
